# Awareness and implementation of tobacco dependence treatment guidelines in Arizona: *Healthcare Systems Survey *2000

**DOI:** 10.1186/1478-4505-6-13

**Published:** 2008-12-19

**Authors:** Mary E Gilles, Louise J Strayer, Robert Leischow, Chun Feng, J Michael Menke, Lee Sechrest

**Affiliations:** 1 The University of Arizona HealthCare Partnership, Department of Psychology, The University of Arizona, Tucson, Arizona, USA; 2 SHPS, Inc, Scottsdale, Arizona, USA

## Abstract

**Background:**

This paper presents findings from the Tobacco Control in Arizona Healthcare Systems Survey, conducted in 2000. The purpose of the survey was to assess the status of Arizona healthcare systems' awareness and implementation of tobacco cessation and prevention measures.

**Methods:**

The 20-item survey was developed by The University of Arizona HealthCare Partnership in collaboration with the Arizona Department of Health Services Bureau of Tobacco Education and Prevention. It was mailed to representatives of Arizona's 40 healthcare systems, including commercial and Medicare managed care organizations, "managed Medicaid" organizations, Veterans Affairs Health Care Systems, and Indian Health Service Medical Centers. Thirty-three healthcare systems (83%) completed the survey.

**Results:**

The majority of healthcare systems reported awareness of at least one tobacco cessation and prevention clinical practice guideline, but only one third reported full guideline implementation. While a majority covered some form of behavioral therapy, less than half reported covering tobacco treatment medications. "Managed Medicaid" organizations administered through the Arizona Health Care Cost Containment System were significantly less likely to offer coverage for behavioral therapy and less likely to cover pharmacotherapy than were their non-Medicaid counterparts in managed care, Veterans Affairs Health Care Systems and Indian Health Service Medical Centers.

**Conclusion:**

Arizona healthcare system coverage for tobacco cessation in the year 2000 was comparable to national survey findings of the same year. The findings that only 10% of "Managed Medicaid" organizations covered tobacco treatment medication and were significantly less likely to cover behavioral therapy were important given the nearly double smoking prevalence among Medicaid patients. Throughout the years of the program, the strategic plan of the Arizona Department of Health Services Bureau of Tobacco Education and Prevention has included the goal of identifying and eliminating tobacco related disparities for special populations, including low-income groups. Of importance, in 2008 the Arizona Health Care Cost Containment System was authorized to provide tobacco cessation pharmacotherapy as a covered benefit for its members.

## Background

In 1995, the Arizona Department of Health Services (ADHS) launched its first comprehensive public Tobacco Education and Prevention Program (TEPP). The name was changed to Bureau of Tobacco Education and Prevention (BTEP) in 2007. Since ADHS-BTEP's inception, Arizona's overall adult smoking rate declined by 23% between 1994 and 2006, from 23.5% to 18.1% [1]. The 2006 prevalence was 13% below the U.S. average of 20.8% for that year [2]. The cost of tobacco dependence is both human and financial: it is the leading preventable cause of death and illness in the United States [3], creating an estimated $96 billion in healthcare expenditures and causing $97 billion in annual productivity losses [4]. In Arizona alone, annual tobacco-related costs exceed $1.3 billion in healthcare expenditures and $1.49 billion in productivity losses [4].

Compared with other preventive disease interventions, tobacco dependence treatment has been shown to be both clinically efficacious and cost-effective [5-7]. Therefore, substantial incentives exist for healthcare systems, health insurers, and health plan purchasers to systematically include tobacco dependence treatment as a covered benefit. Managed care organizations (MCOs) provide a framework for this systematic inclusion with an emphasis on cost-effective treatment. Likewise, purchasers of health plans potentially have the power to influence healthcare delivery by requesting tobacco dependence treatment services and tracking quality of care and health outcomes.

By reducing expenditures for preventable conditions and appealing to purchasers and consumers who value preventive initiatives [8], public health services and tobacco dependence clinical practice guidelines address the needs of MCOs and health plan purchasers alike. Conducted in 2000, the Tobacco Control in Arizona Healthcare Systems Survey arose from the need to clarify and address the relationship among healthcare systems, existing evidence-based guidelines, and public health services for tobacco dependence treatment. The sponsoring organization was The University of Arizona HealthCare Partnership, ADHS-TEPP's continuing education and certification arm, providing planning and evaluation of tobacco dependence treatment outreach programs, and treatment certification for health and human service professionals, health influencers, and local communities statewide.

By the year 2000, Arizona represented one of the most heavily penetrated managed care markets in the United States in both commercial and Medicare MCOs. It was the first state to implement a "managed Medicaid" system, the Arizona Health Care Cost Containment System (AHCCCS). In addition, Arizona has had a traditionally strong tobacco control program and was the first state to establish public smoking bans, in 1972.

Representing the Arizona State Legislature's response to overwhelming county costs for indigent healthcare, AHCCCS was created in 1982 to " [open] up the private physician network to Medicaid recipients and [allow] AHCCCS members to choose a Health Plan and a Primary Care Provider" [9]. Similar to private health insurance, AHCCCS gives patients a choice of health plans that operate on corporate or governmental, for-profit or non-profit bases, depending on the parent organization [9]. AHCCCS capitates monthly payments to its health plans for arrangement of healthcare delivery [10].

The Tobacco Control in Arizona Healthcare Systems Survey was modeled on the series of national surveys first conducted in 1997 as part of the Robert Wood Johnson Foundation's Addressing Tobacco in Managed Care initiative [11]. The Arizona survey shared several objectives with the 1997 national survey, including the assessment of healthcare systems' awareness and practice related to evidence-based tobacco dependence treatment guidelines and the identification of barriers they faced in their efforts to systematically include tobacco dependence treatment [11]. Additionally, the Tobacco Control in Arizona Healthcare Systems Survey aimed to assess healthcare systems' awareness and practice related to tobacco dependence treatment resources, including those offered by ADHS-TEPP. A salient goal for the 2000 survey was to generate baseline data on Arizona healthcare systems for comparison with a projected follow-up survey. Researchers hoped to further elucidate findings related to Arizona's presence as the first managed Medicaid system in the United States and generate comparisons with national data taken at the time.

## Methods

### Survey timing and development

The year 2000 presented an historical time to conduct the Tobacco Control in Arizona Healthcare Systems Survey. This year marked the publication of The Clinical Practice Guideline: *Treating Tobacco Use and Dependence*, released in June 2000 by the U.S. Public Health Service. Other evidence-based guidelines were also available to promote and encourage development of healthcare system policies for tobacco dependence treatment, including the Agency for Healthcare Policy and Research's *Smoking Cessation Clinical Practice Guideline No. 18 *(the 1996 precursor to *Treating Tobacco Use and Dependence*) and evidence-based recommendations developed by the National Cancer Institute and the Veterans Affairs Health Care System. The 2000 Clinical Practice Guideline, in particular, drew from an exhaustive search of the available literature to recommend that all tobacco users receive treatment in accordance with their readiness to quit, that clinicians and healthcare systems standardize the identification and documentation of all tobacco users who present in a healthcare setting, and that health plans and purchasers ensure reimbursement for clinically effective behavioral and pharmacotherapeutic treatments for tobacco dependence [12]. Given the various evidence-based guidelines available at the time, the degree of their awareness and implementation into Arizona healthcare systems was uncertain.

In existence since 1995, the ADHS-TEPP program was well established by 2000. ADHS-TEPP was tasked with bringing the Arizona healthcare community up to speed with the accelerating changes in tobacco dependence treatment. Awareness and implementation of public prevention and treatment resources in 2000 would establish a knowledge base for assessing program progress in subsequent years.

The Master Settlement Agreement (MSA) of 1998 brought into sharp focus the need to monitor delivery of health services for the prevention or treatment of tobacco dependence. Anti-tobacco momentum had reached a threshold whereby state governments needed to take stock of clinical skills and resources available for tobacco dependence treatment.

The 20-item survey was developed by the University of Arizona HealthCare Partnership in conjunction with ADHS-TEPP in early 2000. Health Services Advisory Group, Inc., Arizona's healthcare quality improvement organization, served as a consultant for the survey project. Survey questions addressed the following domains: 1) demographic profile of healthcare systems, including type, enrollment, delivery model, tax status, and accreditation; 2) presence of written tobacco-free workplace policies for employees and/or written tobacco cessation protocols or policies for enrollees; 3) awareness and self-reported implementation of tobacco dependence treatment clinical practice guidelines; 4) awareness of and referral to ADHS-TEPP cessation services; and 5) availability of and coverage for tobacco cessation behavioral interventions and medications for enrollees (Additional File 1).

Other survey questions asked participants to 6) respond to and rank a list of barriers to implementation of tobacco dependence treatment clinical practice guideline recommendations, including such items as "insufficient staff," "cost of implementing guidelines," and "lack of requests from plan purchasers" and 7) respond to and rank a similar list of barriers to provision of tobacco dependence treatment behavioral interventions and medications (Additional File 1).

Not discussed in this article, but included in the scope of the survey, were questions asking participants to 8) describe methods and frequency used to measure enrollee tobacco use; 9) respond to a list of strategies used to motivate clinicians and/or their staff, including such items as "clinician education in tobacco cessation and prevention" and "increased reimbursement for tobacco cessation counseling/assistance"; and 10) identify whether tobacco dependence treatment services were offered as part of a disease management or health promotion program (e.g., asthma or diabetes mellitus).

### Survey implementation

Forty healthcare systems were identified within Arizona, including

• Nine commercial MCOs,

• Eight Medicare MCOs,

• Twelve Arizona Health Care Cost Containment System (AHCCCS) MCOs,

• Three Veterans Affairs (VA) Health Care Systems, and

• Eight Indian Health Service (IHS) Medical Centers.

In the summer of 2000, medical directors of all 40 healthcare systems received a draft survey questionnaire, as well as a letter that apprised them of the project and asked them to identify the appropriate individual within the organization to whom the actual survey should be directed. In addition, the letter explained that all responses from individual healthcare systems would be kept confidential, and that survey reports would reflect aggregate data only. The 20-item questionnaire was then mailed to the medical directors or their designees. Measures to increase response rate included reminder telephone calls, follow-up postcards, and additional questionnaire copies sent to non-respondents after initial contact. Surveys were collected July through September 2000.

### Data analysis

Responses to survey questionnaires were reported in aggregate by healthcare system type. Descriptive analyses of frequency measures and cross-tabulations by type of healthcare system and dominant delivery model were compared. Fisher's exact test was used to test whether AHCCCS MCOs differed significantly from non-AHCCCS commercial and Medicare MCOs in providing behavioral therapy including and beyond self-help.

## Results

Of the 40 healthcare systems contacted, 33 (83%) responded to the survey. These included 7 commercial, 7 Medicare, 10 AHCCCS MCOs, 3 VA Health Care Systems, and 6 IHS Medical Centers. Response rates by healthcare system type were 82% for commercial and Medicare MCOs, 83% for AHCCCS MCOs, 100% for VA Health Care Systems, and 75% for IHS medical centers. Surveys were completed most frequently by a Health Care Quality Manager (24%); other responders included Medical Directors (15%), Clinical Directors (9%), and Health Educators (9%).

### Awareness and implementation of guideline recommendations

Among responding healthcare systems, 88% – including 100% of non-AHCCCS MCOs, VA Health Care Systems, and IHS Medical Centers – reported awareness of at least one clinical practice guideline for tobacco dependence treatment (Table 1). Two AHCCCS MCOs, representing 20% of AHCCCS respondents and 6% of total respondents, reported that they were not aware of any guidelines, while an additional two AHCCCS MCOs (20%) did not respond to the question.

**Table 1:** Awareness and implementation of tobacco dependence treatment guidelines by healthcare system type

	**Arizona Health Care Cost Containment System (AHCCCS) MCO**	**Non-AHCCCS MCO (Commercial & Medicare)**	**Veterans Affairs (VA)**	**Indian Health Service (IHS)**	**All Healthcare Systems**
					
	**(Total = 10)**	**(Total = 14)**	**(Total = 3)**	**(Total = 6)**	**(Total = 33)**
**Awareness of Guidelines**	**N(%)**	**N(%)**	**N(%)**	**N(%)**	**N(%)**

Yes	6(60)	14(100)	3(100)	6(100)	29(88)

No	2(20)	0	0	0	2(6)

No Response	2(20)	0	0	0	2(6)

**Implementation of Guidelines**					

Full	1(10)	4(29)	2(67)	4(67)	11(33)

Partial	1(10)	5(36)	1(33)	0	7(21)

No	6(60)	3(21)	0	0	9(27)

No Response	2(20)	2(14)	0	2(33)	6(18)

Thirty-three percent of all healthcare systems reported full implementation of at least one clinical practice guideline, including 10% of AHCCCS MCOs, 29% of non-AHCCCS MCOs, 67% of VA Health Care Systems, and 67% of IHS Medical Centers. Twenty-one percent reported partial implementation, including 10% of AHCCCS MCOs, 36% of non-AHCCCS MCOs, and 33% of VA healthcare systems. Eighteen percent of all responding healthcare systems reported no implementation of guidelines: 60% of the AHCCCS MCOs and 21% of the commercial and Medicare MCOs.

**Figure 1:**
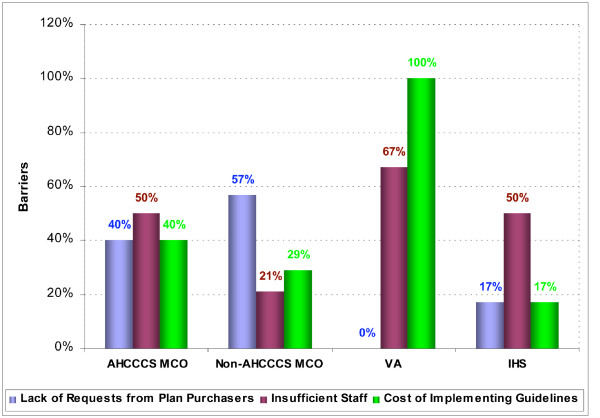
**Most frequently listed barriers to implementation of tobacco dependence treatment guidelines by healthcare system type**.

Healthcare systems reported various barriers to implementation of tobacco dependence treatment guideline recommendations (Figure 1). The three most frequently listed barriers were "lack of requests from plan purchasers," "insufficient staff," and "cost of implementing guidelines." Reported barriers varied by healthcare system type; for example, "cost of implementing guidelines" was cited by 100% of responding VA Health Care Systems, while "insufficient staff" was cited by 50% of AHCCCS MCOs and IHS Medical Centers. "Lack of requests from plan purchasers" was cited by 57% of non-AHCCCS MCOs.

### Reimbursement coverage of tobacco dependence treatment interventions

Eighty-two percent of all healthcare systems reported coverage of at least one form of behavioral therapy for tobacco dependence treatment, including self-help materials (Table 2). This percentage fell to 70% for behavioral therapy beyond self-help, as 12% of all healthcare systems provided self-help materials only. AHCCCS MCOs were significantly less likely to provide coverage for any form of behavioral therapy including self-help, as well as for behavioral therapy beyond self-help, than non-AHCCCS MCOs. In addition, 30% of AHCCCS MCOs reported lack of coverage for any behavioral therapy for tobacco dependence treatment.

**Table 2:** Coverage of tobacco dependence treatment services by healthcare system type

**Coverage for Behavioral Services**	**AHCCCS MCO**	**Non-AHCCCS MCO (Commercial & Medicare)**	**VA**	**IHS**	**All Healthcare Systems**	**P Value (Fisher's Exact Test)**
	**(Total = 10)** **N(%)**	**(Total = 14)** **N(%)**	**(Total = 3)** **N(%)**	**(Total = 6)** **N(%)**	**(Total = 33)** **N(%)**	***AHCCCS vs. non-AHCCCS***
**Behavioral Therapy** **Including Self-Help**	5(50)	14(100)	3(100)	5(83)	27(82)	p = 0.006

**Behavioral Therapy** **Beyond Self-Help**	3(30)	12(86)	3(100)	5(83)	23(70)	p = 0.008

**Self-Help Materials Only**	2(20)	2(14)	0	0	4(12)	

No	3(30)	0	0	0	3(9)	

No Response	1(10)	0	0	1(17)	2(6)	

**Medication Coverage**						

Yes	1(10)	5(36)	3(100)	5(83)	14(42)	

No	9(90)	7(50)	0	0	16(48)	

No Response	0	2(14)	0	1(17)	4(9)	

Less than half of all responding healthcare systems (42%) reported coverage of at least one tobacco dependence treatment medication (Table 2). One hundred percent of VA Health Care Systems and 83% of IHS Medical Centers provided medication coverage, as compared to 36% of non-AHCCCS MCOs and 10% of AHCCCS MCOs.

**Figure 2:**
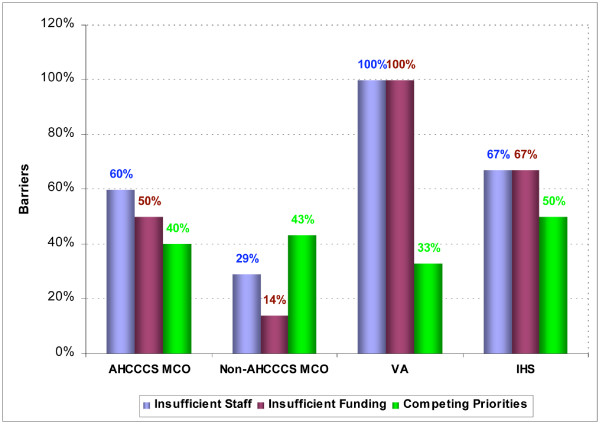
**Most frequently listed barriers to provision of tobacco dependence treatment interventions by healthcare system type**.

The three most frequently cited barriers to provision of tobacco dependence treatment interventions, such as coverage for behavioral therapies and medication, were "insufficient staff," "insufficient funding," and "competing priorities" (Figure 2). As with implementation of practice guidelines, barriers varied by healthcare system type; however, resource-related barriers predominated. One hundred percent of VA Health Care Systems cited "insufficient staff" and "insufficient funding" as barriers, while 67% of IHS Medical Centers cited these items as barriers. "Insufficient staff" was also cited by 60% of AHCCCS MCOs, followed closely by "insufficient funding" at 50%. A lower percentage of non-AHCCCS MCOs, however, cited these items as barriers.

### Awareness of state tobacco dependence treatment resources

A high percentage of healthcare systems were aware of or referred patients to the free tobacco dependence treatment services offered through ADHS-TEPP, including the Arizona Smokers' Helpline (a statewide tobacco quitline) and local county health department projects, as well as tribal projects and Indian Health Service initiatives throughout the state. Fifty-eight percent of healthcare systems reported referring patients to the Arizona Smokers' Helpline, while an additional 12% were aware of the Helpline but did not refer patients to it. Non-AHCCCS (79%) and AHCCCS (70%) MCOs were more likely to refer patients to the Helpline than were VA Health Care Systems (0%) and IHS Medical Centers (17%). Forty-five percent of healthcare systems reported referring patients to ADHS-TEPP-funded community-based projects offering tobacco dependence treatment services, and an additional 21% reported awareness of these projects. Again, non-AHCCCS (57%) and AHCCCS (50%) MCOs were more likely to refer patients to local projects than were VA Health Care Systems (0%) and IHS Medical Centers (33%).

## Discussion

### A tale of two surveys

The results of the Arizona Healthcare Systems 2000 survey were compared with the Addressing Tobacco in Managed Care (ATMC) survey executed in 2000 [13]. Both surveys collected and analyzed data addressing implementation of tobacco cessation guidelines, provision of behavioral therapy and pharmacotherapy for tobacco cessation, and barriers to addressing tobacco dependence in healthcare systems. The ATMC surveyed a sample of 85 national health insurance plans. The ATMC survey did not include VA Health Care Systems or IHS Medical Centers. Results of the ATMC survey showed that 56.5% of plans had implemented clinical practice guidelines for tobacco dependence treatment. Eighty-six percent covered some form of behavioral therapy and 69% allowed members to self-refer to behavioral therapy. Over half (59.2%) of the national plans fully covered at least one type of tobacco dependence treatment medication, ranging from 13.3% for over-the-counter and prescription nicotine replacement therapies to 44% for bupropion SR, a non-nicotine prescription medication. Approximately three-fourths of national plans *required *providers to ask new patients about smoking status, 68.3% to deliver "strong advice" to quit, and 36.5% to arrange follow-up visits with patients making quit attempts.

The 2000 ATMC survey and the Arizona Healthcare Systems survey results were comparable in response rates and findings (Table 3). In fact, no significant differences were found in response rate, degree of implementation, and coverage for behavioral therapy and tobacco dependence treatment medication, as tested by χ^2 ^test for proportions. Though awareness of clinical practice guidelines was not measured *per se *in the national survey, it could be inferred that national awareness was at least as high as in Arizona, given the similar extent of self-reported guideline implementation as reported nationally.

**Table 3:** Results of the 2000 Arizona survey compared with the 2000 national survey

		**National Survey**	**Arizona Survey**	**Comparison**	
		*(McPhillips-Tangum et al., 2002)*	*(Gilles et al., 2008)*		
				Chi Square	Significance Level

**Size of Initial Mail-Out**		136	40		

**Response Rate**		62.5%	83.0%	0.029	0.865

**Awareness of Guidelines**		NA	88.0%	NA	NA

**Implementation of Guidelines**		56.5%	54.0%	0.001	0.981

**Full Coverage**	At Least One Tobacco Treatment Medication	59.2%	42.0%	0.029	0.866
	
	At Least One Behavioral Intervention	86.2%	70.0%	0.017	0.897

### Context and future plans

The survey was undertaken during an important time in tobacco control history. Arizona, the site of the first-ever public smoking bans in 1972, lagged slightly but not significantly behind the U.S. as a whole in terms of behavioral therapy and medication coverage in the year 2000. More importantly, Arizona's "Managed Medicaid" organizations appeared to lag behind commercial MCOs for coverage of behavioral therapy and tobacco dependence treatment medication, even though tobacco use is higher among the medically indigent. (For instance, the 2005 national smoking rate for Medicaid patients was 36%, versus 20.6% for the general population [12,13].) Specifically, 10% of AHCCCS MCOs offered pharmacotherapy aids for tobacco dependence treatment, though up to 40% of their enrollees smoke. This finding suggests a misdirection of resources at that time.

According to subsequent ATMC surveys [14], health insurance coverage for tobacco dependence treatment has grown since 2000, but has leveled off in recent years. Health plans that pay for either behavioral therapy or tobacco treatment medication reached a maximum at 98% in 2000, and then decreased slightly to 96.2% in 2003. Face-to-face counseling reimbursement dropped from 41.1% to 35.9% between 2000 and 2003, though this difference probably does not suggest a significant change in direction or policy.

Several recent developments in Arizona's tobacco control landscape represent major policy changes that may affect the future of tobacco dependence treatment throughout the state. In April 2008, the Arizona Department of Health Services Bureau of Tobacco Education and Prevention issued its new strategic plan, which targets at-risk populations with tailored media messages directing tobacco users to the Quitline and other prevention and treatment resources [15]. For the foreseeable future, ADHS-BTEP's strategic goals also include more targeted use of the Internet, text messaging, and other tools to reach vulnerable populations.

Another positive development in 2008 provides coverage for Medicaid enrollees seeking pharmacotherapeutic assistance with tobacco cessation. On April 29, 2008, Arizona Senate Bill 1418 was signed into law, authorizing the Medicaid program to cover tobacco dependence treatment medications for eligible members [16]. This is intended to reduce the impact of tobacco-related illness in a population with high tobacco-use prevalence. Currently, the AHCCCS Medicaid program spend $316 million each year – 14% of its annual costs – on tobacco-related illness [17].

## Conclusion

The 2000 survey was a timely assessment and inventory of the stock and status of tobacco control resources within Arizona healthcare systems. Cigarette taxes and price increases in 2002 and 2006, followed by a statewide smoking ban in 2007, and a deluge of media coverage on all fronts of tobacco control, have all likely stimulated continued interest in quitting tobacco use. Whether the current resources needed for tobacco dependency are met by healthcare system resources is a topic left to future research. The two new frontiers for Arizona in tobacco dependence treatment are likely to be further treatment for the medically indigent and primary prevention of tobacco use in vulnerable youth.

## Competing interests

MEG is a member of the Speaker's Bureau for Pfizer, Inc.

## Authors' contributions

MEG conceived of the study, participated in its design and coordination, and led the drafting of the manuscript. LJS, LS, and RL participated in the design and execution of the study. JMM and CF performed the statistical analysis and drafted the manuscript. All authors read and approved the final manuscript.

## Supplementary Material

Additional file 1 Survey questions discussed in this article. Click here for file
