# Supplementary material for: Awareness and implementation of tobacco dependence treatment guidelines in Arizona: Healthcare Systems Survey 2000
Source: Health Res Policy Syst. 2008 Dec 19;6:13. doi: 10.1186/1478-4505-6-13 (PMC2628749; doi:10.1186/1478-4505-6-13)
Supplement: Additional file 1 — Survey questions discussed in this article. [file 1478-4505-6-13-S1.pdf]

Additional File 1. Survey questions discussed in this article

Survey Question:	
**Which type of healthcare system do you represent? (If you represent more than one type, please request an additional survey)**	☐ MCO Commercial<br>☐ MCO Medicare<br>☐ MCO AHCCCS<br>☐ VA<br>☐ IHS<br>☐ other (specify)
**Which of the following best describes the total enrollment of your Arizona healthcare system membership? (check only one)**	☐ Less than 25,000<br>☐ 25,001 to 50,000<br>☐ 50,001 to 100,000<br>☐ 100,001 to 250,000<br>☐ 250,001 to 500,000<br>☐ More than 500,000
**What percent of your healthcare system enrollment receives its care through the following delivery models? (your answers should total 100%)**	___% Group<br>___% Staff<br>___% Independent Practice Association<br>___% Direct Contract<br>___% Network<br>___% Other (specify)
**What is the tax status of your healthcare system?**	☐ For profit<br>☐ Not for profit
**Please indicate which if any of the following accreditations are held by your healthcare system. (check all that apply)**	☐ NCQA (National Committee for Quality Assurance)<br>☐ JCAHO (Joint Commission on Accreditation of Healthcare Orgs.)<br>☐ URAC (Utilization Review Accreditation Commission)<br>☐ Other (specify)<br>☐ None
**Does your healthcare system collect and/or report HEDIS (Health Employer Data and Information Set) data? (check only one)**	☐ Collects<br>☐ Collects and reports<br>☐ Reports<br>☐ Neither collects nor reports
**Does your healthcare system have an in-house tobacco-free workplace policy for <u>employees</u>?**	☐ Yes<br>☐ No
**Is your healthcare system aware of tobacco cessation and prevention guidelines? (mark all that apply)**	☐ AHCPR (Agency for Health Care Policy & Research)<br>___ No<br>___ Yes<br>___ If yes, please indicate implementation (full, partial, none)<br>☐ US Public Health Service<br>___ No<br>___ Yes

	___ If yes, please indicate implementation (full, partial, none)<br>☐ NCI (National Cancer Institute)<br>___ No<br>___ Yes<br>___ If yes, please indicate implementation (full, partial, none)<br>☐ VA (Veterans' Affairs)<br>___ No<br>___ Yes<br>___ If yes, please indicate implementation (full, partial, none)<br>☐ Other (Specify)<br>___ No<br>___ Yes<br>___ If yes, please indicate implementation (full, partial, none)
**Does your healthcare system have a written tobacco cessation protocol/policy for <u>enrollees</u>?**	☐ Yes<br>☐ No
**Which of the following barriers limit the feasibility of fully implementing tobacco cessation and prevention <u>guidelines or policies</u> in your healthcare system? (mark all that apply and rank the top three barriers with 1 being the greatest barrier)**	**Barrier Rank**<br>☐ ___ Insufficient staff<br>☐ ___ Cost of implementing guidelines<br>☐ ___ No evidence for cost-effectiveness of guidelines<br>☐ ___ Limited access to tobacco cessation programs<br>☐ ___ Takes too much time away from patient care duties by clinicians<br>☐ ___ Guidelines conflict with internally developed or pre-existing tobacco cessation & prevention guidelines<br>☐ ___ Lack of scientific validity/efficacy of guidelines<br>☐ ___ Lack of support from upper management<br>☐ ___ Lack of requests from plan purchasers<br>☐ ___ Other (specify)
**Please indicate which of the following outside resources in Arizona your healthcare system is aware of and refers members to. (mark all that apply)**	☐ Arizona Smokers' Helpline<br>___ Not aware<br>___ Aware<br>___ Refers to<br>☐ Arizona Department of Health Services Tobacco Education and Prevention Program Local Projects<br>___ Not aware<br>___ Aware<br>___ Refers to<br>☐ Other (specify)<br>___ Not aware<br>___ Aware<br>___ Refers to
**Indicate the status of the following tobacco cessation interventions to**	☐ Telephone Counseling<br>___ Covered for general enrollment

**your enrollees.**	___ Covered for some contracts<br>___ Not covered<br>___ Not available<br>☐ Face-to-Face Counseling<br>___ Covered for general enrollment<br>___ Covered for some contracts<br>___ Not covered<br>___ Not available<br>☐ Group Counseling<br>___ Covered for general enrollment<br>___ Covered for some contracts<br>___ Not covered<br>___ Not available<br>☐ Self-help Materials<br>___ Covered for general enrollment<br>___ Covered for some contracts<br>___ Not covered<br>___ Not available<br>☐ Other (Specify)<br>___ Covered for general enrollment<br>___ Covered for some contracts<br>___ Not covered<br>___ Not available
**Indicate the coverage for the following tobacco cessation medications to your enrollees.**	☐ Nicotine gum<br>___ Covered for general enrollment<br>___ Covered for some contracts<br>___ Covered with cessation program<br>___ Not covered<br>___ On formulary<br>___ Paid for OTC<br>☐ Nicotine patches<br>___ Covered for general enrollment<br>___ Covered for some contracts<br>___ Covered with cessation program<br>___ Not covered<br>___ On formulary<br>___ Paid for OTC<br>☐ Nicotine nasal spray<br>___ Covered for general enrollment<br>___ Covered for some contracts<br>___ Covered with cessation program<br>___ Not covered<br>___ On formulary<br>☐ Nicotine inhaler

	___ Covered for general enrollment<br>___ Covered for some contracts<br>___ Covered with cessation program<br>___ Not covered<br>___ On formulary<br>☐ Bupropion<br>___ Covered for general enrollment<br>___ Covered for some contracts<br>___ Covered with cessation program<br>___ Not covered<br>___ On formulary
**Please identify the barriers that most limited your healthcare system's ability to provide tobacco <u>interventions</u> to your members. (mark all that apply and rank the top three barriers with 1 being the greatest barrier)**	**Barrier Rank**<br>☐ ___ Insufficient funding<br>☐ ___ Insufficient resources for clinician training<br>☐ ___ Competing priorities<br>☐ ___ Inability to identify tobacco users<br>☐ ___ Referral systems not in place<br>☐ ___ Insufficient staff<br>☐ ___ Lack of evidence of cost-effectiveness of interventions<br>☐ ___ Limited access to tobacco cessation programs<br>☐ ___ Takes too much time away from other clinician requirements for patient care<br>☐ ___ Lack of continuity of enrollment of members<br>☐ ___ Lack of scientific evidence validity/efficacy of interventions<br>☐ ___ Lack of requests from health plan purchasers<br>☐ ___ Low participation rates by members<br>☐ ___ Low member demand for services<br>☐ ___ Other (specify)
